# Role of rs4986790 Polymorphism of *TLR4* Gene in Susceptibility towards Malaria Infection in the Pakistani Population

**Published:** 2018-05

**Authors:** Asima RANI, Syed Kashif NAWAZ, Muhammad ARSHAD, Shazia IRFAN

**Affiliations:** 1. Dept. of Zoology, University of Sargodha, Sargodha, Pakistan; 2. Dept. of Biological Sciences, University of Sargodha, Sub Campus, Mianwali, Pakistan; 3. Principal, University of Education, Lower Mall Campus, Lahore, Pakistan

**Keywords:** TLR4, rs4986790, Malaria, *Plasmodium*, Pakistani population

## Abstract

**Background:**

Toll-like receptors (TLRs) of the human immune mechanism play important role in the detection of invading pathogens. TLRs specifically recognize the pathogen-associated molecular patterns (PAMPs) from pathogens and start the effective response. Single nucleotide polymorphisms (SNPs) in the TLRs can mediate their functions. Present study evaluated the importance of rs4986790 polymorphism of *TLR4* gene in susceptibility towards malaria, clinical outcomes of the disease and responsible species of malaria.

**Methods:**

Blood samples of 228 malaria patients and 226 healthy volunteers were selected for the study. Sample collection was completed during Sep 2013 to Sep 2015 from different hospitals of Punjab, Pakistan. Patient’s samples were divided into *P. vivax* group and *P. falciparum* group on the basis of causative species of *Plasmodium*. Malaria samples were also divided into mild and severe malaria group based on clinical outcomes of the disease according to WHO criteria. Healthy individuals were placed in the control group. Whole blood was used for the isolation of DNA. Genomic DNA was isolated and amplification of targeted SNP was performed using allele-specific PCR.

**Results:**

Results indicate the protective role of AA genotype against the susceptibility of *P. vivax* infection, OR: 0.5, 95%CI: 0.285–0.876, *P*=0.038.

**Conclusion:**

rs4986790 polymorphism of *TLR4* gene modulates the susceptibility towards *P. vivax* infection. AA genotype is found to be protective against the development of *P. vivax* infection in the local population of Pakistan.

## Introduction

Immune system protects against various infections through the detection of invading pathogens and subsequent activation of inflammatory response. Malaria is an infectious disease of the tropical and subtropical world caused by protozoan parasite, *Plasmodium.* Malaria is transmitted through bite of an infectious *Anopheles* mosquito. Almost 60% of the Pakistani population lives in malaria-endemic regions of the country. Two species of *Plasmodium* were considered as the main agents for malaria infection in Pakistan; *P. falciparum* and *P. vivax* (1, 2).

Toll-like receptors (TLRs) present on the surface of immune cells recognize PAMPs (pathogen-associated molecular patterns) from pathogens. Upon recognition of PAMPs, specific immune response is initiated. *Plasmodium* parasite induces various responses in the host (1). However specific parasitic ligands and their specific receptors in host as well as signaling liable for host immune response remains controversial (1). Almost 10 TLRs have been recognized so far in human beings, specific for various PAMP’s. TLR2 specifically recognizes lipoproteins, *TLR4* recognizes lipopolysaccharides (LPS), TLR3, 4, 5, 6, 7 and 8 recognize nucleotides associated with pathogens (3). These receptors initiate inflammatory response in the host. Inflammatory response is beneficial for host and helps in parasitic clearance. *TLR4* specifically recognize lipopolysaccharides (LPS). TLR 4 is an important receptor involved in the recognition of *Plasmodium*. It has been reported to identify glycosylphosphatidylinositol (GPI) from the *Plasmodium* parasite. Dysregulation in TLR signaling affects the inflammatory response and may contribute in development of chronic inflammatory diseases (4). Defects in the identification of parasite or activation of receptor can both results in severe clinical outcomes of the disease (4).

Association of polymorphisms in certain TLRs with susceptibility to malaria has not been confirmed yet. Various factors like ethnicity, environment, pathogen, and host render it difficult to infer a certain association between susceptibility to malaria and TLRs polymorphisms. Various studies on different human populations will help to clarify the problem (5).

Human *TLR4* gene is located on chromosome 9, locus 9q32–q33. *TLR4* consists of 4 exons and show maximum expression in lymphocytes, monocytes, neutrophils, and splenocytes (6). Single nucleotide polymorphisms (SNPs) affecting the *TLR4* function modulates the development and severity of infectious diseases. Two non-synonymous polymorphisms in *TLR4* gene were studied in association with different diseases, rs4986790 and rs4986791.

SNP in the fourth exon of TLR4, rs4986790 causes substitution of adenine (A) to guanine (G) at 896bp region. This substitution results in amino acid replacement of aspartic acid with glycine at position 299 (Asp299Gly). It has been reported to cause disruption in the extracellular domain of *TLR4* due to the presence of G allele. This allele alters the extracellular domain of this receptor and disturbs the transport of *TLR4* to cell membrane. This change alters the ability of host to respond towards various pathogens (7). This variation reduces *TLR4* signaling and *TLR4* becomes unable to efficiently activate inflammatory response. This polymorphism results in diminished response of *TLR4* to LPS and thus less production of inflammatory cytokines (8).

The mutant allele G has been reported in association with reduced *TLR4* signaling, dampened inflammatory response, lower levels of soluble adhesion molecules and acute phase reactants. The carriers of G allele were found hypo-responsive to inhaled LPS (7,9). Various studies reported the association of wild allele A with enhanced activation of *TLR4* and thus increased levels of inflammatory cytokines. Association of A allele has been reported in inflammatory diseases like type 2 diabetes and atherosclerosis (10,11).

Malaria can develop into severe disease. Rather than the species of *Plasmodium*, other factors may also determine the disease severity and progression including the genotype of host. Specific receptors for pathogens, their activation and signaling may decide the disease development and outcome. This study considers the hypothesis that polymorphism in *TLR4* gene can modulate the disease susceptibility and clinical outcome of malaria. Presence of A allele or AA homozygosity may develop enhanced inflammatory response, while G allele or GG homozygous individuals may develop attenuated inflammatory response. Both exaggerated and attenuated inflammatory response can develop severe disease outcomes. Enhanced inflammatory response can cause tissue damage, while reduced response is unable to clear the parasitic load. Heterozygous individuals may develop balanced inflammatory response, thus causing mild disease outcomes.

## Materials and Methods

Ethical consideration: All procedures were according to the declaration of Helsinki. The advanced research and study board University of Sargodha approved the study protocol. Prior permission from ethical committee, University of Sargodha was also taken to start the study.

Criteria for sample selection: Human whole blood samples of 516 individuals were collected for the study. Gender and age-matched controls and cases were selected for the study. Among the collected samples, 454 samples were successfully amplified and selected for the study, whereas sixty-two samples could not be genotyped due to the failure in PCR amplification. The study involves 228 malaria samples and 226 healthy controls.

Blood collection: The patient’s blood was collected from various hospitals of Punjab, Pakistan during Sep 2013 to Sep 2015. Blood samples (5ml, venous blood) were collected in EDTA vials and stored at −20 °C till analysis.

Identification of malaria samples: Patients were confirmed on the basis of presence/absence of *Plasmodium* parasite in the blood. *Plasmodium* parasite was detected via kit method (ImuMed, China). Kit method involves addition of 5ul blood sample into the sample well (S) of test cassette, followed by the addition of 3 drops of lysis buffer in well B. After 30 min the results were calculated. If line appears on control and *Pv* then there is *P. vivax* in the sample. If line appears on control and *Pf* then there is *P. falciparum* in the sample. If line only appears on control, then there is no *Plasmodium* species or malaria. Patient’s samples were divided into two groups based on causative species of *Plasmodium; P. falciparum* and *P. vivax*.

Criteria for severe and mild malaria groups: The malaria samples were also divided into severe malaria and mild malaria groups according to WHO criteria. Severe malaria-infected patients have neurological problems (prostration, lethargy), severe anemia (Ht<20%, Hb<6 g/dl), hyperparasitemia, gastrointestinal symptoms, hypoglycemia (serum glucose less than 40 mg/dl), oliguria, acidosis with respiratory distress, jaundice, cardiovascular shock and diffuse hemorrhages. Rests of the samples were placed in mild malaria group.

Blood samples of healthy volunteers were collected as controls from the local population. Amplification of rs4986790 polymorphism of *TLR4* gene: Genomic DNA was isolated from whole blood using vivantis blood DNA isolation kit (Cat# GF-BD-100). Allele-specific PCR technique was used for the amplification of rs4986790. Two forward primers F1: 5′CTACTACCTCGATGA3′, F2: 5′CTACTACCTCGATGG3′ and one reverse primer R: 5′ CAGCCATTTTCAAGA3′ was used for amplification reaction. PCR involves initial step of denaturation (94°C for 5 min), followed by 30 cycles of denaturation (94°C for 30 sec), annealing (45.3°C for 30 sec) and extension (68°C for 30 sec). Final extension was performed at 68°C for 12 min.

Detection of genotypes: Agarose gel electrophoresis following UV illumination was used for the detection of results. Agarose gel of 0.8% was used for DNA detection and 2% was used for the detection of PCR product. PCR product of 557bp with F1primer was read as AA, with F2 primer was read as GG and with both F1 and F2 primers was read as AG genotype. Samples, which gave amplification results only with F1 primer, depict the presence of A allele and AA homozygous sample. Samples, which gave amplification results only with F2 primer, show the presence of G allele and GG homozygous sample. Samples, which gave amplification results with both F1 and F2 primers, indicate the presence of both A and G alleles and AG heterozygous sample. PCR product was detected in comparison with the DNA Ladder (Invitrogen, cat. no.: 10416-014) that was run on agarose gel.

### Statistical Analysis

Hardy Weinberg Equilibrium (HWE) estimation and genetic frequencies, allelic frequencies, and differences in the frequencies were analyzed using Chi-square. SPSS software, ver. 18 for Windows (SPSS Inc., Chicago Illinois, USA) was used for analysis of Chi-square test and other nonparametric tests. The relationships of various genotypes with malaria groups were examined using odds ratio (OR) considering control group as the reference. Odds ratios were performed using an online calculator (12).

## Results

Table 1 depicts the characteristics of malaria patients and healthy controls. Mean age of patients was 22.67±13.64 yr and a healthy control was 22.24±2.75 yr. Gender-matched controls were selected for the study.

**Table 1:** Characteristics of malaria patients and healthy controls

***Groups***	***Age (yr) (Mean±SD)***	***Gender (Male%)***	***SM***	***MM***	***P. falciparum malaria***	***P. vivax malaria***
Malaria (N=228)	22.67±13.64	53.5%	89	139	100	128
Control (N=226)	22.24±2.75	55.3%	00	00	00	00
*P*-value	0.64	0.70	ND^*^	ND^*^	ND^*^	ND^*^

Student t-test was performed for comparison of means, SM=severe malaria, MM=mild malaria, ND=not determined, *P*-value=statistical *P*-value

* Due to sample size zero, *P*-value is not determined

Table 2 presents the results of genotype and allele frequencies of rs4986790 in various malaria groups, control group, and overall population. Frequency of A allele was higher in all groups as compared to G allele. GG homozygous samples were not observed in the *P. falciparum* group. Results of HWE estimation indicated that all groups (*P*<0.05) were deviant from HWE except *P. falciparum* group.

**Table 2:** Genotype and allele frequencies of rs4986790 polymorphism of TLR4 gene in different malaria groups and controls with results of HWE

***Genotypic and allele frequencies with results of HWE in malaria groups***							
***Genotypes and alleles***	***Groups on the basis of symptoms***		***Groups on the basis of parasite***		***Total Malaria (N=228)***	***Control (N=226)***	***Total samples (N=454)***
	***MM (N=139)***	***SM (N=89)***	***P. falciparum (N=100)***	***P. vivax (N=128)***			
AA N(Freq)	114(0.82)	78(0.88)	94(0.94)	98(0.76)	192(0.84)	196(0.87)	388(0.86)
AG N(Freq)	21(0.15)	9(0.10)	6(0.06)	24(0.19)	30(0.13)	26(0.11)	56(0.12)
GG N(Freq)	4(0.03)	2(0.02)	0(0)	6(0.05)	6(0.03)	4(0.02)	10(0.02)
A N(Freq)	125(0.9)	82(0.93)	97(0.97)	110(0.86)	207(0.91)	208(0.92)	415(0.92)
G N(Freq)	14(0.1)	7(0.07)	3(0.03)	18(0.14)	21(0.09)	18(0.08)	39(0.08)
HWE (*P*)	5.1 (0.023)	5.7 (0.017)	0.1 (0.751)	6.44 (0.011)	10.37 (0.001)	6.77 (0.009)	17.41 (0.000)

SM=severe malaria, MM=mild malaria, HWE - Hardy Weinberg equilibrium, (*P*) = statistical *P* value, N=no. of individuals, Freq=frequency

Table 3 depicts the results of association of rs4986790 with malaria groups. This polymorphism had no significant association with susceptibility to malaria, mild malaria and severe malaria. Association of genotypes and malaria groups indicated, that AA genotype has protective effect towards susceptibility to *P. vivax* malaria. It showed 0.5 times decrease in the chances of *P. vivax* malaria (OR: 0.5, 95%CI: 0.285–0.876, *P*=0.038). AG and GG genotypes had positive but marginal association with susceptibility to *P. vivax* malaria. AG genotype increased 1.775 times (OR: 1.775, 95%CI: 0.971–3.245, *P*=0.038) and GG genotype increased 2.729 times the chances of *P. vivax* malaria (OR: 2.729, 95%CI: 0.755–9.859, *P*=0.03). rs4986790 polymorphism did not depict any association with *P. falciparum* malaria. Association of malaria groups and alleles did not show any significant association with malaria groups.

**Table 3:** Association of rs4986790 polymorphism of TLR4 gene with malaria groups

***Groups***	***Association of malaria groups and genotypes***				***Association of malaria groups and alleles***		
	**AA**	**AG**	**GG**	**χ^2^ (p)**	**A**	**G**	**χ^2^ (p)**
Malaria OR (95% CI)	0.816 (0.483–1.378)	1.165 (0.665–2.041)	1.5 (0.417–5.388)	0.718 (0.69)	0.853 (0.441–1.647)	1.172 (0.606–2.264)	0.224 (0.636)
MM OR (95% CI)	0.698 (0.391–1.245)	1.369 (0.737–2.540)	1.644 (0.404–6.684)	1.575 (0.454)	0. 772 (0.371–1.607)	1.294 (0.621–2.693)	0.478 (0.489)
SM OR (95% CI)	1.085 (0.518–2.272)	0.865 (0.388–1.928)	1.275 (0.229– 7.092)	0.194 (0.907)	1.182 (0.45–3.08)	0.845 (0.32–2.2)	0.118 (0.731)
*P. falciparum* OR (95% CI)	2.398 (0.964–5.959)	0.491 (0.195–1.233)	---	4.322 (0.115)	2.798 (0.805–9.725)	0.357 (0.102–1.242)	2.835 (0.092)
*P. vivax* OR (95% CI)	0.5 (0.285–0.876)	1.775 (0.971–3.245)	2.729 (0.755– 9.859)	6.516 (0.038)	0.528 (0.264–1.057)	1.890 (0.945–3.781)	3.326 (0.068)

For calculation of odds ratios (ORs), each group was compared with the control group, CI=confidence interval, SM=severe malaria, MM=mild malaria, χ^2^ =Chi square, (*P*)=statistical *P* value.

## Discussion

Some malaria-infected individuals develop severe malaria, while others only suffer from mild or uncomplicated malaria. This variation in infection response and disease progression may develop due to various environmental and genetic variations. Variations in immunity, age, genetic makeup of host and parasite may determine the clinical outcomes of disease. Resistance to infection mainly depends on the immune system of the host. Innate immune mechanism of humans has TLRs, which specifically recognizes various pathogens through PAMPs. These TLRs initiate the intracellular signaling which starts the expression of various effector molecules that mediates the adaptive immunity. TLR genes have polymorphic nature; genetic variations in these genes can affect the pathogenesis of different diseases (13–15).

Genetic polymorphism in TLRs genes was reported to have association with different diseases like age-related macular degeneration (AMD), Alzheimer, atherosclerosis, and prostate cancer (16,17).

SNPs in the TLR2, *TLR4* and TLR9 polymorphisms have been studied for altering the susceptibility and resistance to various inflammatory and infectious diseases (8). SNPs in the TLRs genes and their signaling partners were supposed to mediate the susceptibility towards malaria and its clinical outcome (18).

The results indicate the higher frequency of A allele as compared with G allele. This is in accordance with the NCBI data; the global allele frequency of A allele is higher as compared with G allele (NCBI) (19). We did not find any association between the studied polymorphism and the susceptibility of malaria. The rs4986790 polymorphism can mediate the susceptibility/resistance to malaria infection by differential activation of *TLR4* (8). Already available literature also shows the association of rs4986790 polymorphism with susceptibility to malaria (5, 20, 21). However, we did not find any association between the studied polymorphism and malaria susceptibility in the Pakistani population.

We did not find the association of this polymorphism with severe or mild malaria. These results are in accordance (22, 23). The protective effect of GG genotype of rs4986790 was reported against severe malaria (21). This polymorphism is associated with low parasitemia and mild forms of malaria. The association of GG genotype of rs4986790 polymorphism was reported with mild and uncomplicated malaria (5, 20). G allele of rs4986790 protects against development of severe malaria (24). However, GG genotype of rs4986790 polymorphism increased the risk of severe pediatrics malaria in the Ghana population (25).

Our results indicate the protective role of AA genotype of rs4986790 in the susceptibility of *P. vivax* infection. G allele of rs4986790 has been associated with reduced inflammatory response to lipopolysaccharide (7). A allele has been reported to result in the enhanced inflammatory response, which may help in the removal of *P. vivax* load in the studied population and protects from *P. vivax* infection. The presence of A allele could increase the chances of *TLR4* activation and thus increased inflammatory response and atherosclerosis (10). These different results may indicate the different disease mechanism of both diseases. Enhanced inflammatory response can increase the atherosclerosis development, however, in infectious diseases, inflammatory response helps in the parasitic clearance. Marginal risk association between *P. falciparum* and rs4986790 of *TLR4* gene cannot exclude the importance of *TLR4* gene, as other polymorphisms in the *TLR4* gene may mediate the *TLR4* response to *P. falciparum*.

This is the first study which relates the association between rs4986790 polymorphism of *TLR4* gene and malaria in the Pakistani population. This study helps intellectuals in considering the importance of this polymorphism in pathogenesis of various diseases. Study of other polymorphisms in the *TLR4* gene, association between inflammatory and anti-inflammatory cytokines with rs4986790 genotypes in malaria groups and meta-analysis may investigate the role of rs4986790 polymorphism in the severity of malaria.

## Conclusion

rs4986790 polymorphism of *TLR4* gene can modulate the susceptibility towards *P. vivax* infection. AA genotype is found to be protective against the development of *P. vivax* infection in the local population of Pakistan.

## Ethical considerations

Ethical issues (Including plagiarism, informed consent, misconduct, data fabrication and/or falsification, double publication and/or submission, redundancy, etc.) have been completely observed by the authors.
